# High vimentin expression with E-cadherin expression loss predicts a poor prognosis after resection of grade 1 and 2 pancreatic neuroendocrine tumors

**DOI:** 10.1186/s12885-021-08062-6

**Published:** 2021-03-31

**Authors:** Bo Zhou, Jie Xiang, Ming Jin, Xiang Zheng, Guogang Li, Sheng Yan

**Affiliations:** 1 Department of Hepatobiliary and Pancreatic Surgery, The Second Affiliated Hospital, Zhejiang University School of Medicine, Hangzhou, 310003 China; 2 Department of Hepatobiliary and Pancreatic Surgery, The First Affiliated Hospital, School of Medicine, Zhejiang University, Hangzhou, China

**Keywords:** Pancreatic neuroendocrine tumors, Epithelial-mesenchymal transition, Vimentin, E-cadherin, Prognosis

## Abstract

**Background:**

Pancreatic neuroendocrine tumors (pNETs) are a heterogeneous group of neoplasms with malignant behaviors that can develop from inert slow growth or low malignancy to aggressive metastasis during follow-up. Recently, vimentin and E-cadherin were shown to be prognostic markers in some malignant tumors but were not evaluated in pNETs. The aim of this study was to evaluate the expression and prognostic significance of vimentin and E-cadherin in grade 1 and 2 pNETs.

**Methods:**

A retrospective review of 227 patients with grade 1 and 2 pNETs undergoing surgical resection was conducted. Tumor specimens were immunohistochemically stained for vimentin and E-cadherin. Correlations between vimentin and E-cadherin expression and other clinicopathological features were then analyzed. Furthermore, overall survival (OS) and disease-free survival (DFS) were evaluated using Kaplan-Meier and univariate and multivariate Cox regression methods.

**Results:**

Among 227 patients, 55 (24.2%) harbored tumors with high vimentin expression, while 117 (51.5%) harbored tumors with loss of E-cadherin expression. Patients with high vimentin expression and loss of E-cadherin expression had significantly elevated risks of lymph node metastasis, distant metastasis, perineural invasion and an advanced American Joint Committee on Cancer (AJCC) stage compared with those with low vimentin expression and preserved E-cadherin expression, high vimentin expression and preserved E-cadherin expression, or low vimentin expression and loss of E-cadherin expression. Furthermore, multivariate analysis showed that high vimentin expression with loss of E-cadherin expression was an independent predictor of OS and DFS in patients with grade 1 and 2 pNETs who underwent resection (both *P* < 0.001).

**Conclusions:**

The current study demonstrated that high vimentin expression with loss of E-cadherin expression was correlated with lymph node metastasis, distant metastasis, disease progression and a poor prognosis in patients with grade 1 and 2 pNETs who underwent resection.

## Background

Pancreatic neuroendocrine tumors (pNETs) are a rare heterogeneous group of neoplasms that represent approximately 2% of all pancreatic neoplasms [1, 2]. Advances in imaging technologies have improved the detection rate and diagnosis of this rare pancreatic disease [3]. pNETs can be subdivided into functional and nonfunctional cases. The majority of pNET cases (60–90%) are nonfunctional [4]. Compared with pancreatic ductal adenocarcinoma, pNETs have better long-term survival rates [5, 6]. Recently, several large cohort studies found that lymph node involvement, tumor stage and grade, tumor size and Ki-67 index scores could predict the prognosis of pNETs [5–7]. However, some patients with small tumors or tumors with a low World Health Organization (WHO) grade (grade 1 or 2) may exhibit liver or lymph node metastasis and have a poor prognosis [8]. Little is known about the molecular and cellular mechanisms involved in the invasion and metastasis of pNETs.

Cancer cell invasion of surrounding normal tissues is a critical step in the invasion and metastasis of malignant disease [9]. Increasing evidence indicates that epithelial-mesenchymal transition (EMT) plays critical roles in tumor development and metastasis [10–12]. In cancer progression and metastasis, cancer cells destroy or degrade the extracellular matrix, migrate and invade other tissues. Moreover, several EMT transcription factors, such as Snail, Slug, Twist, and Zeb, ultimately promote epithelial inhibition and mesenchymal feature induction [12]. Epithelial-cadherin (E-cadherin), a cell-surface adhesion molecule, is essential in maintaining apical-basal polarity in epithelial cells [13]. The expression of E-cadherin decreases during EMT in the processes of embryonic development, tissue repair and cancer metastasis. Vimentin, a hallmark of EMT, is involved in cellular motility, cell shape maintenance and directional migration [14]. It was reported that acquisition of vimentin expression and loss of E-cadherin expression during EMT results in tumor metastasis, invasion, radioresistance and generation of cancer cells with stem cell-like characteristics in pancreatic cancer [15].

With the aim of gaining insight into the molecular and clinical mechanisms underlying metastatic progression, the present study evaluated the expression of vimentin and E-cadherin and their effects on the progression and prognosis of patients with grade 1 and 2 pNETs who underwent surgery.

## Methods

### Study population

The medical data of patients with grade 1 and 2 pNETs undergoing surgical resection between January 2007 and November 2019 at the Second Affiliated Hospital and First Affiliated Hospital of Zhejiang University School of Medicine were retrospectively reviewed. A diagnosis of pNETs was made using standard histological criteria. The following clinicopathological data were collected for the patients: age; sex; symptoms at presentation; tumor location; type of surgical procedure; pathological characteristics including tumor size, lymph node involvement, perineural invasion and Ki-67 index; and stage. This study was approved by the Ethics Review Committee of the Second Affiliated Hospital of Zhejiang University School of Medicine.

### Immunohistochemistry

Immunohistochemical staining was performed using a standard avidin-biotin-peroxidase complex procedure. Briefly, five-micrometer-thick sections were cut and subjected to antigen retrieval using citrate buffer (pH = 6.0). The sections were then incubated with primary antibodies (anti-vimentin (ab8978) at 1:200, anti-E-cadherin (ab1416) at 1:100, Abcam Inc., Cambridge, UK) overnight at 4 °C, followed by incubation with a secondary antibody for 1 h at room temperature. The immunohistochemical reaction was visualized with 3,3 diaminobenzidine (DAB) as the substrate.

All tissue sections were simultaneously assessed by two investigators in a blinded manner. The immunohistochemical signal intensity was scored using the following scale: 0 for background staining, 1 for faint staining, 2 for moderate staining, and 3 for strong staining. In addition, the percentage of positive cells was graded according to the following scale: 0 for < 5%, 1 for 5–25%, 2 for 26–50%, 3 for 50–75%, and 4 for 75–100%. These two parameters were multiplied to produce a weighted score for each specimen; 0–2 and ≥ 3 were considered negative and positive staining, respectively.

### Follow-up

Clinical follow-up was conducted by reviewing hospital medical records or via telephone. Patients were usually evaluated at our department every 6 months for the first 5 years after surgery and once a year thereafter. The postoperative follow-up data collected included clinical symptoms and signs, laboratory test results and imaging examination findings. Overall survival (OS) was defined as the time interval between the date of surgery and the date of death or last follow-up. Disease-free survival (DFS) was defined as the period from the date of surgery to the date of tumor recurrence or last follow-up.

### Statistical analysis

Results are presented as the mean ± standard derivation (SD), and all statistical analyses were performed using SPSS 26.0 software (IBM Corp., Armonk, NY, USA). The associations between EMT markers (vimentin and E-cadherin) and other prognostic factors were analyzed using the chi-square test or Fisher’s exact test. The Kaplan-Meier method and log-rank test were used to calculate and compare OS and DFS. Univariate and multivariate Cox regression models were performed to analyze prognostic factors. A *P*-value < 0.05 was considered statistically significant.

## Results

### Patient characteristics

A total of 227 patients were included in the present analysis. These patients were diagnosed at a mean age of 52.48 ± 13.55 years and were evaluated over a mean follow-up period of 69.66 ± 35.49 months. Of the 227 patients with pNETs, 94 (41.4%) had their disease detected incidentally during physical examination. The most common symptom was abdominal pain (*n* = 64), which was frequently accompanied by hypoglycemia, abdominal discomfort and jaundice. The median size of pNETs was 2.5 (range, 0.4–19.0) cm. The most common tumor location in the pancreas was the head (*n* = 78, 47.1%), followed by the neck (*n* = 25, 36.6%) and then body and tail (*n* = 124, 16.3%). Among the patients, 222 underwent curative resection (R0 resection, 97.8%), while the others received palliative surgery (R1 resection, 2.2%). The surgical procedures included distal pancreatectomy (*n* = 108), pancreaticoduodenectomy (*n* = 72), enucleation (*n* = 30), middle pancreatectomy (*n* = 16), and total pancreatectomy (*n* = 1). Lymph node metastasis was confirmed pathologically in 24 (10.6%) patients. One hundred seventeen (51.5%) of the patients had grade 1 disease, and the remaining 110 (48.5%) had grade 2 disease. The majority of patients (193/227, 85.0%) had stage I or II disease.

At the time of the last follow-up, 34 patients had relapsed, and 16 patients had died. For the entire population, the 1-, 3- and 5-y OS rates were 99, 95 and 92%, respectively, and the 1-, 3- and 5-y DFS rates were 94, 86 and 85%, respectively.

### Associations between vimentin/E-cadherin expression and other clinicopathologic parameters in pNETs

**Fig. 1:**
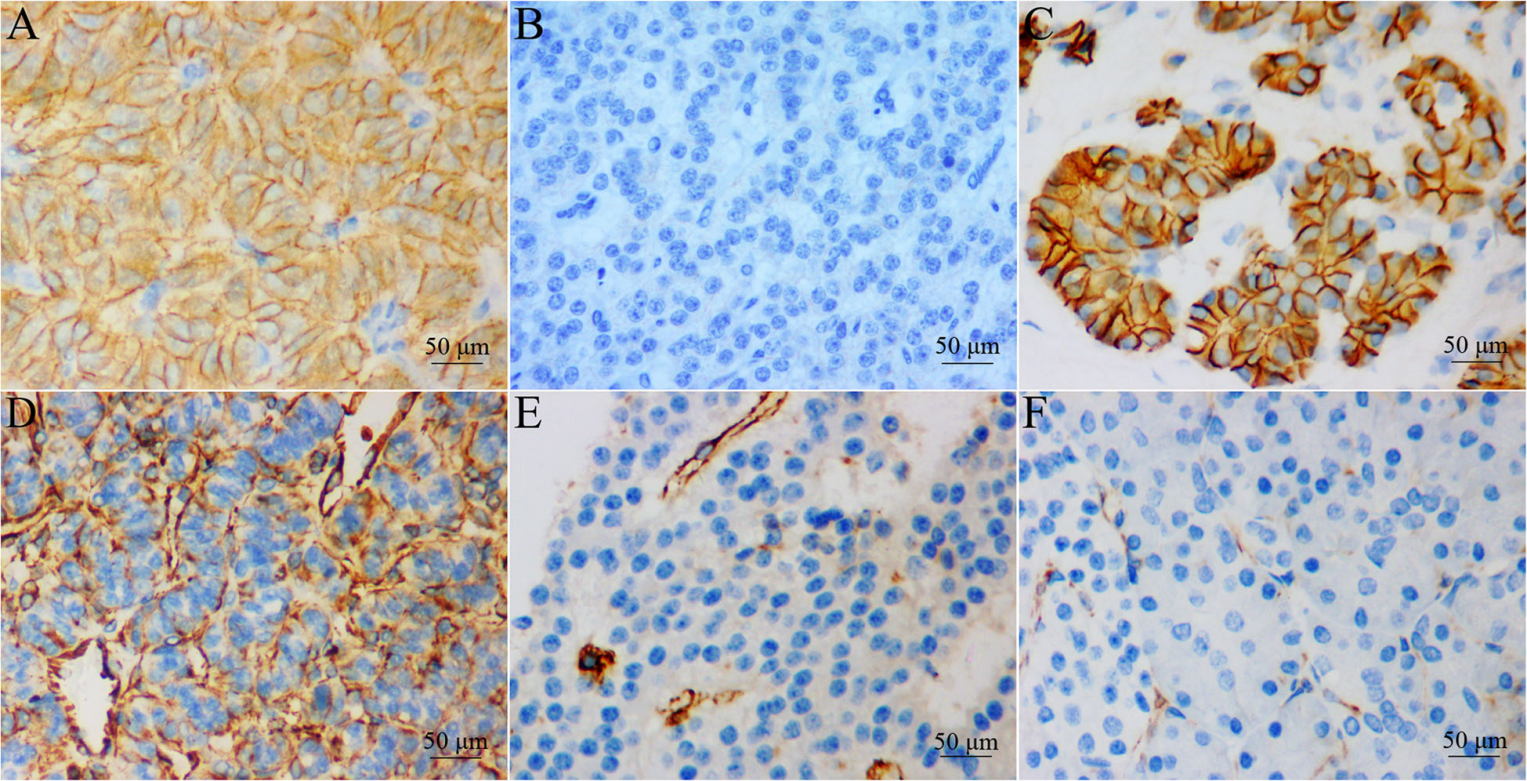
Representative images of E-cadherin and vimentin expression in pancreatic neuroendocrine tumors and normal pancreatic tissues (400×). **a** Intense membranous expression of E-cadherin in pNETs. **b** Loss of E-cadherin in pNETs. **c** E-cadherin expression in normal pancreatic tissue. **d** Strong cytoplasmic expression of vimentin in pNETs. **e** Low cytoplasmic expression of vimentin in pNETs. (F) Vimentin expression in normal pancreatic tissue

E-cadherin expression was identified in the membrane of pNET cells, while vimentin expression was identified in the cytoplasm of pNET cells (Fig. 1). Preserved E-cadherin expression was observed in 110 patients with pNETs (48.0%) (Fig. 1a), while loss of E-cadherin expression was observed in 117 patients with pNETs (Fig. 1b). In the 227 patients, high and low vimentin expression levels were observed in 55 (24.2%) and 172 (75.8%) patients, respectively (Fig. 1d and e). Furthermore, vimentin expression was more frequently observed in tumors with lymph node (LN) metastasis (*P* < 0.001), distant metastasis (*P* = 0.002), a larger size (*P* = 0.044), perineural invasion (*P* = 0.023), a higher WHO grade (*P* = 0.010) or an advanced American Joint Committee on Cancer (AJCC) stage (*P* < 0.001) (Table 1). Similar to the results for vimentin, loss of E-cadherin expression was more common in tumors with a higher T-stage (*P* = 0.026), LN metastasis (*P* = 0.015), distant metastasis (*P* = 0.022), a larger size (*P* = 0.003), perineural invasion (*P* = 0.007), a higher WHO grade (*P* < 0.001) or an advanced AJCC stage (*P* = 0.002) (Table 1). No significant difference were observed in terms of sex, age, tumor location or symptoms with respect to the expression of vimentin or E-cadherin.

**Table 1:** Associations between vimentin/E-cadherin expression and other clinicopathological factors in pNETs

Variable	Total, n	Vimentin expression, n			E-cadherin expression, n		
		Low	High	*P*	Lost	Preserved	*P*
Age, years				0.205			0.908
≤ 60	156	122	34		80	76	
> 60	71	50	21		37	34	
Sex				0.239			0.902
Male	100	72	28		52	48	
Female	127	100	27		65	62	
Symptoms				0.944			0.696
Absent	94	71	23		47	47	
Present	133	101	32		70	63	
Tumor size, cm				0.044			0.003
≤ 2	101	83	18		41	60	
> 2	126	89	37		76	50	
Tumor location				0.344			0.578
Head/uncinate/neck	103	75	28		51	52	
Body/tail	124	97	27		66	58	
T-stage				0.538			0.026
T1–2	180	138	42		86	94	
T3–4	47	34	13		31	16	
LN metastasis				< 0.001			0.015
Absent	203	161	42		99	104	
Present	24	11	13		18	6	
Distant metastasis				0.002			0.022
Absent	218	169	49		109	109	
Present	9	3	6		8	1	
Perineural invasion				0.023			0.007
Absent	204	159	45		99	105	
Present	23	13	10		18	5	
WHO classification				0.010			< 0.001
Grade 1	117	97	20		45	72	
Grade 2	110	75	35		72	38	
AJCC stage				< 0.001			0.002
I-II	193	156	37		91	102	
III-IV	34	16	18		26	8	

*pNETs* Pancreatic neuroendocrine tumors, *LN* Lymph node, *AJCC* American Joint Committee on Cancer

As acquisition of vimentin and loss of E-cadherin expression during EMT contribute to tumor invasiveness and metastatic capacity in cancer, we divided all patients into two groups as follows: the high vimentin and lost E-cadherin expression group (*n* = 42) and the ‘other group’ (patients with low vimentin and preserved E-cadherin expression, high vimentin and preserved E-cadherin expression, or low vimentin and lost E-cadherin expression) (Table 2). Tumors with high vimentin and lost E-cadherin expression were more common in patients with LN metastasis (*P* < 0.001), distant metastasis (*P* = 0.003), perineural invasion (*P* = 0.034) and an advanced AJCC stage (*P* < 0.001). Additionally, 1/117 patients with G1 tumors exhibited distant metastasis at diagnosis, while 6/117 patients with G1 tumors relapsed. The tumor tissues of these patients showed high vimentin and lost E-cadherin protein expression.

**Table 2:** Associations between vimentin and E-cadherin coexpression and other clinicopathological factors in pNETs

Variable	Total, n	Vimentin and E-cadherin expression pattern, n		
		High vimentin and lost E-cadherin	Other	*P*
Age, years				0.291
≤ 60	156	26	130	
> 60	71	16	55	
Sex				0.390
Male	100	21	79	
Female	127	21	106	
Symptoms				0.407
Absent	94	15	79	
Present	133	27	106	
Tumor size, cm				0.107
≤ 2	101	14	87	
> 2	126	28	98	
Tumor location				0.505
Head/uncinate/neck	103	21	82	
Body/tail	124	21	103	
T-stage				0.769
T1–2	180	34	146	
T3–4	47	8	39	
LN metastasis				< 0.001
Absent	203	30	173	
Present	24	12	12	
Distant metastasis				0.003
Absent	218	37	181	
Present	9	5	4	
Perineural invasion				0.034
Absent	204	34	170	
Present	23	8	15	
WHO classification				0.111
Grade 1	117	17	100	
Grade 2	110	25	85	
AJCC stage				< 0.001
I-II	193	26	167	
III-IV	34	16	18	

*pNETs* Pancreatic neuroendocrine tumors, *LN* Lymph node, *AJCC* American Joint Committee on Cancer

### Prognostic significance of vimentin and E-cadherin expression

First, we performed survival analysis according to E-cadherin alone or vimentin alone. The Kaplan-Meier method was used to analyze patient survival, and the data showed that the 1-, 3- and 5-year OS rates of the patients with low vimentin expression were markedly higher than those with high vimentin expression (100, 99.4 and 98.7% vs 96.3, 81.3 and 72.8%, respectively, *P* < 0.001), and the 1-, 3- and 5-year DFS rates of the patients with low vimentin expression were also significantly higher than those with high vimentin expression (97.7, 95 and 94.2% vs 81.6, 58.3 and 58.3%, respectively, *P* < 0.001). The 1-, 3- and 5-year OS rates of the patients with preserved E-cadherin expression were markedly higher than those with loss of E-cadherin expression (100, 98.1 and 98.1% vs 98.3, 90.9 and 85.2%, respectively, *P* < 0.001), and the 1-, 3- and 5-year DFS rates of the patients with preserved E-cadherin were also significantly higher than those with loss of E-cadherin expression (97.2, 96.3 and 96.3% vs 90.5, 76 and 74.5%, respectively, *P* < 0.001).

**Fig. 2:**
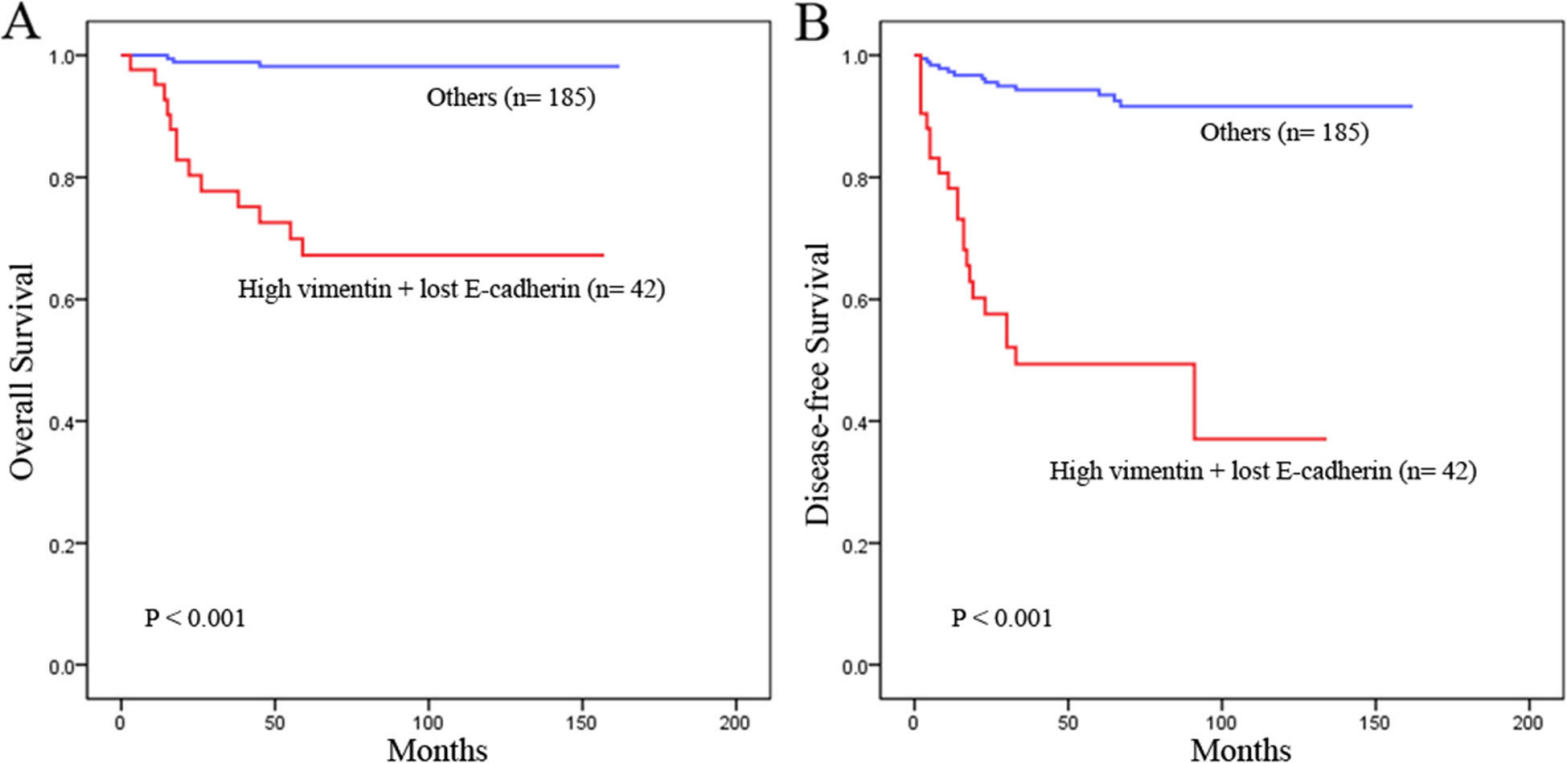
Kaplan-Meier survival curves showing OS (**a**) and DFS (**b**) for patients who underwent surgery for pNETs stratified by vimentin and E-cadherin coexpression

Furthermore, the 1-, 3- and 5-year OS rates of the high vimentin and lost E-cadherin expression group were markedly lower than those of the other group (95.2, 77.7 and 67.2% vs 99.4, 98.9 and 98.2%, respectively, *P* < 0.001) (Fig. 2a), and the 1-, 3- and 5-year DFS rates of the high vimentin and lost E-cadherin expression group were also significantly lower than those of the other group (78.2, 49.4 and 49.4% vs 97.3, 94.3 and 93.5%, respectively, *P* < 0.001) (Fig. 2b). Therefore, our research showed that high vimentin expression with loss of E-cadherin expression during EMT was correlated with poor patient survival after resection of pNETs.

The results from the univariate analyses of clinicopathological variables are shown in Table 3. High vimentin with lost E-cadherin expression (*P* < 0.001), LN metastasis (*P* < 0.001), symptoms (*P* = 0.039), perineural invasion (*P* = 0.019), distant metastasis (*P* < 0.001), WHO classification (*P* = 0.010) and AJCC stage (*P* < 0.001) were prognostic factors for OS. Similarly, the significant predictors of DFS were high vimentin with lost E-cadherin expression (*P* < 0.001), sex (*P* = 0.040), tumor size (*P* = 0.032), symptoms (*P* = 0.039), perineural invasion (*P* = 0.030), T-stage (*P* = 0.039), distant metastasis (*P* < 0.001), LN metastasis (*P* < 0.001), WHO classification (*P* < 0.001) and AJCC stage (*P* < 0.001).

**Table 3:** Variables associated with OS according to Cox proportional hazards regression models

Variables	Univariate analysis			Multivariate analysis		
	HR	95% CI	*P*	HR	95% CI	*P*
Sex			0.106			
Male	Reference					
Female	NA	NA				
Age, years			0.097			
≤ 60	Reference					
> 60	NA	NA				
Tumor size, cm			0.575			
≤ 2	Reference					
> 2	NA	NA				
Symptoms			0.039			0.168
Absent	Reference			Reference		
Present	4.768	1.084–20.982		NA	NA	
Tumor location			0.424			
Head/uncinate/neck	Reference					
Body/tail	NA	NA				
T-stage			0.12			
T1–2	Reference					
T3–4	NA	NA				
Perineural invasion			0.019			0.430
Absent	Reference			Reference		
Present	3.870	1.246–12.023		NA	NA	
LN metastasis			< 0.001			0.784
Absent	Reference			Reference		
Present	11.916	4.433–32.028		NA	NA	
Distant metastasis			< 0.001			0.690
Absent	Reference			Reference		
Present	10.102	3.252–31.380		NA	NA	
WHO classification			0.010			0.540
Grade 1	Reference			Reference		
Grade 2	5.253	1.496–18.443		NA	NA	
AJCC stage			< 0.001			< 0.001
I-II	Reference			Reference		
III-IV	18.602	5.996–57.709		8.646	2.645–28.261	
EMT markers			< 0.001			< 0.001
Other group	Reference			Reference		
High vimentin and lost E-cadherin expression group	20.888	5.951–73.318		10.388	2.794–38.625	

*OS* Overall survival, *pNETs* Pancreatic neuroendocrine tumors, *LN* Lymph node, *AJCC* American Joint Committee on Cancer, *EMT* Epithelial-mesenchymal transition

By multivariate analysis, high vimentin with lost E-cadherin expression and AJCC stage were found to be independent predictive factors for OS (both *P* < 0.05) (Table 3), whereas high vimentin with lost E-cadherin expression, distant metastasis, AJCC stage and WHO classification were independent predictors for DFS (all *P* < 0.05) (Table 4).

**Table 4:** Variables associated with DFS according to Cox proportional hazards regression models

Variables	Univariate analysis			Multivariate analysis		
	HR	95% CI	*P*	HR	95% CI	*P*
Sex			0.040			0.735
Male	Reference			Reference		
Female	2.052	1.035–4.068		NA	NA	
Age, years			0.146			
≤ 60	Reference					
> 60	NA	NA				
Symptoms			0.039			0.287
Absent	Reference			Reference		
Present	2.302	1.042–5.086		NA	NA	
Tumor size, cm			0.032			0.572
≤ 2	Reference			Reference		
> 2	2.306	1.076–4.940		NA	NA	
Tumor location			0.869			
Head/uncinate/neck	Reference					
Body/tail	NA	NA				
T-stage			0.039			0.498
T1–2	Reference			Reference		
T3–4	2.102	1.040–4.249		NA	NA	
Perineural invasion			0.030			0.331
Absent	Reference			Reference		
Present	2.665	1.099–6.462		NA	NA	
LN metastasis			< 0.001			0.390
Absent	Reference			Reference		
Present	5.787	2.857–11.724		NA	NA	
Distant metastasis			< 0.001			< 0.001
Absent	Reference			Reference		
Present	25.166	11.112–56.996		6.295	2.385–16.617	
WHO classification			< 0.001			0.003
Grade 1	Reference			Reference		
Grade 2	6.014	2.487–14.541		4.106	1.601–10.528	
AJCC stage			< 0.001			0.004
I-II	Reference			Reference		
III-IV	11.475	5.773–22.811		3.474	1.485–8.126	
EMT markers			< 0.001			< 0.001
Other group	Reference			Reference		
High vimentin and lost E-cadherin	9.836	4.904–19.724		6.565	3.180–13.553	

*DFS* Disease-free survival, *pNETs* Pancreatic neuroendocrine tumors, *LN* Lymph node, *AJCC* American Joint Committee on Cancer, *EMT* Epithelial-mesenchymal transition

## Discussion

In the current study, we showed that the analysis of vimentin and E-cadherin coexpression had prognostic importance in patients with grade 1 and 2 pNETs. pNETs in the high vimentin and lost E-cadherin expression group and the ‘other group’ were considered to have mesenchymal and epithelial phenotypes, respectively. We observed that pNETs with a mesenchymal phenotype frequently had LN metastasis, distant metastasis, perineural invasion and an advanced AJCC stage. Furthermore, high vimentin with lost E-cadherin expression was an independent predictive factor for OS and DFS in patients with grade 1 and 2 pNETs who underwent resection.

In recent years, increasing molecular data for pNETs have accumulated [16, 17]. However, the genetic basis of pNET progression and metastasis remains unclear. EMT refers to a mechanism whereby tumor cells acquire the features of mesenchymal cells [18]. During EMT, cancer cells lose the ability to recognize specific targets and usually develop autocrine loops for growth factors, which play essential roles in providing self-sustaining growth signals to cancer cells [19]. E-cadherin, which is expressed by epithelial cells, plays a crucial role in cell-cell contact. Loss or downregulation of E-cadherin expression, a critical event in EMT, results in the subsequent progression of cells towards a malignant phenotype [20–22]. Vimentin, a mesenchymal marker, can play fundamental roles in tumor invasion and metastasis. It has been reported that high vimentin and reduced E-cadherin expression is an important predictor in various types of cancers, such as gastric cancer, colorectal cancer and pancreatic cancer [23–25].

To date, a few studies have been conducted to evaluate the expression of EMT-specific transcription factors in neuroendocrine tumors [26–28]. Fendrich et al. immunohistochemically evaluated the expression of the EMT factors Snail, Twist and E-cadherin in human pNETs and showed overexpression of Snail and Twist and loss of E-cadherin expression in the majority of malignant pNETs [26], which was similar to the results of Yonemori K [29]. However, no statistically significant correlation was found between Snail/Twist expression or loss of E-cadherin expression and OS, but there was an inverse association between E-cadherin and Snail/Twist. Regarding pulmonary neuroendocrine tumors, Hwang W et al. showed that EMT could contribute to EGFR-tyrosine kinase inhibitor (TKI) resistance [30]. In our study, we found that grade 1 and 2 pNETs with high vimentin and lost E-cadherin expression were significantly associated with LN metastasis, distant metastasis, perineural invasion, an advanced AJCC stage and a poor prognosis.

This study has several potential limitations that must be considered. First, the study was retrospective in design and was subject to selection bias and diagnostic bias. Second, our sample may not be generally representative because all the patients included in this study were Chinese. Additionally, only patients with grade 1 and 2 pNETs undergoing surgical resection were included in the study, so this work does not cover most advanced cases; thus, our results may not apply to patients without indications for surgical resection due to an advanced stage. Despite these limitations, the present study is valuable because it identifies vimentin and E-cadherin as potential prognostic markers to predict the survival of patients with pNETs undergoing surgical resection.

## Conclusions

The current study demonstrated that high vimentin and loss of E-cadherin expression were correlated with LN metastasis, distant metastasis, disease progression and a poor prognosis in patients with grade 1 and 2 pNETs who underwent resection.

## Data Availability

The datasets used and analyzed in our study are available from the corresponding authors upon reasonable request.

## Abbreviations

E-cadherin: Epithelial-cadherin
pNET: Pancreatic neuroendocrine tumor
EMT: Epithelial-mesenchymal transition
WHO: World Health Organization
AJCC: American Joint Committee on Cancer
OS: Overall survival
DFS: Disease-free survival
SD: Standard derivation
LN: Lymph node
